# The stress-inducible ER chaperone GRP78/BiP is upregulated during SARS-CoV-2 infection and acts as a pro-viral protein

**DOI:** 10.1038/s41467-022-34065-3

**Published:** 2022-11-14

**Authors:** Woo-Jin Shin, Dat P. Ha, Keigo Machida, Amy S. Lee

**Affiliations:** 1 Florida Research and Innovation Center, Cleveland Clinic, Port St. Lucie, FL 34987 USA; 2 Department of Biochemistry and Molecular Medicine, Keck School of Medicine, University of Southern California, Los Angeles, CA 90033 USA; 3 Norris Comprehensive Cancer Center, Keck School of Medicine, University of Southern California, Los Angeles, CA 90033 USA; 4 Department of Molecular Microbiology and Immunology, Keck School of Medicine, University of Southern California, Los Angeles, CA 90033 USA

**Keywords:** Mechanisms of disease, SARS-CoV-2

**arising from** M.S. Shaban et al. *Nature Communications* 10.1038/s41467-021-25551-1 (2021)

The current Coronavirus global pandemic necessitates the search for novel targets and therapeutic avenues. Shaban et al.^1^ reported the use of an endoplasmic reticulum (ER) stress inducer thapsigargin (Tg) to block replication of Coronaviruses (CoVs), and that a key mechanism for the anti-viral activities of Tg is to counteract virus-mediated GRP78/BiP downregulation. This conclusion contradicts the widely observed upregulation of GRP78 by virus infection and the reported pro-viral roles of GRP78^2,3^. In contrast to their findings which were based primarily on short term culture of infected cells, here we have documented the temporal increase of both GRP78 mRNA and protein levels as SARS-CoV-2 infection intensifies, and that GRP78 knockdown or inhibition of its activity suppresses SARS-CoV-2 replication and infectivity in vitro and in vivo, implying that GRP78 could be a valuable stable host target to combat COVID-19.

The molecular chaperone GRP78/BiP or HSPA5 that in humans is encoded by the *HSPA5* gene, and has recently been identified as a host auxiliary factor for SARS-CoV-2 entry^4^. For simplicity, this protein is referred to as GRP78 thereafter. Importantly, computer modeling including structural dynamics and binding analysis reveals that host-cell recognition through GRP78 is enhanced in the new variants of SARS-CoV-2 associated with increased transmissibility^5,6^. The study by Shaban et al.^1^ that was published in *Nature Communications* explored the use of the ER stress inducer Tg, a well-established inducer of GRP78, to block replication of CoVs by counteracting CoV-mediated suppression of IRE1α and GRP78. The authors concluded that Tg induced an anti-viral state by blocking the CoV-induced autophagic flux and reactivating the ER quality control (ERQC) or ER-associated degradation (ERAD) network of proteins that were suppressed by infection of CoVs. Furthermore, the authors stated that CoVs suppressed GRP78 expression in infected cells and Tg-induced GRP78 re-expression is a major mechanism for the anti-viral activities of Tg. While the anti-viral effects of Tg on multiple different CoVs in different cell types are clearly demonstrated by Shaban et al.^1^, in view of the emerging implications of GRP78 in enhancing SARS-CoV-2 and its variants for entry and subsequent function in viral protein production and infectivity^2–4^, some mechanistic explanations offered for the Tg effects based on the short-term culture of infected cells may need further consideration.

First, the broad contention by the authors that infection by CoVs suppressed GRP78 expression in the host cells is concerning, as there may be differences between endemically adapted virus such as HCoV-229E versus highly pathogenic novel human CoVs such as SARS-CoV-2. In Shaban et al.^1^, the authors primarily examined cells up to 24 hr post-infection (hpi). While a statistically significant suppressive effect on GRP78 expression was evident in the HCoV-229E virus-infected Huh7 cells (Fig. 2e, f in ref.^1^), infection with HCoV-229E did not seem to decrease GRP78 protein level in the MRC-5 cells (Fig. 4b, lane 3 in ref.^1^), and it is difficult to draw conclusions on whether SARS-CoV-2 negatively affected basal GRP78 expression in the cell lines examined in the absence of pharmacologically-induced stress by Tg (Fig. 4g, h, lanes 1,4,7,10 and Fig. 9a in ref. 1).

The assertion that infection by CoVs led to a suppression of GRP78 by the authors is not consistent with most observations about the important roles of GRP78 in viral infections including CoVs in literature to date^2,3^. As the authors pointed out in the study, viral infection has been widely known to cause ER stress and activate the unfolded protein response (UPR). As an essential ER chaperone and master regulator of the UPR, GRP78 is upregulated during ER stress and generally accepted as a canonical marker of ER stress induction and UPR activation^7,8^. Viruses are obligate intracellular parasites that are dependent on cellular machinery to replicate their genome and produce viral proteins for the assembly of progeny virions. Enveloped viruses such as CoVs are heavily dependent on the ER membrane and machinery to manufacture viral proteins and derive their viral envelop membrane. Thus, GRP78 has been established as a pro-viral protein for many different virus infections including CoVs^2,3^. For instance, GRP78 has been found to be important for SARS-CoV-2, MERS-CoV, and bCoV-HKU9 entry since blocking the cell surface form of GRP78 by specific antibodies inhibited viral entry and infection^4,9^. Chu et al.^9^ further showed that GRP78 was upregulated on the surface of MERS-CoV-infected Huh7 cells at 24 hpi. Higher GRP78 levels were observed in Vero E6 cells with SARS-CoV-2 infection as revealed by immunofluorescence staining and blocking GRP78 activity by the small molecule inhibitor AR12 suppressed SARS-CoV-2 replication^10^. In another study, SARS-CoV-2 infection elevated GRP78 protein level in Calu-3 cells at 24 hpi and interestingly, SARS-CoV-2 infection slightly suppressed GRP78 level in Vero CCL81 cells at 24 hpi but subsequently enhanced GRP78 level at 48 hpi^11^. In primary human lung microvascular endothelial cells, MERS-CoV infection led to a substantial increase in GRP78 protein level at 24 hpi^12^. Importantly, autopsy analysis of lungs from COVID-19 patients and non-COVID-19 controls showed that pneumocytes from SARS-CoV-2-infected lungs exhibited robust in situ GRP78 immunostaining compared with that from uninfected controls^13^. Serum GRP78 levels were also reported to be significantly higher in patients during COVID-19 infection compared to control groups^14^.

Given that Shaban et al.^1^ only examined infection of CoVs up to 24 hpi, it is tempting to speculate that they might have missed the subsequent induction of GRP78 as virus infection intensifies. With a focus on SARS-CoV-2, the causative agent of the current pandemic, we examined the kinetics of SARS-CoV-2 infection on GRP78 protein levels in Vero E6-ACE2 cells at an MOI of 0.5, following experimental conditions used in Shaban et al.^1^. We further repeated the experiments in a human lung epithelial cell line H1299. The cells were incubated at 33 ^o^C in a CO_2_ incubator and collected at 12, 24, 36, and 48 hpi, and cell lysates were analyzed by Western blot for GRP78 protein levels, with viral Spike protein and GAPDH serving as viral infection and loading control respectively (Fig. 1a–c). The specificity of the antibody for detection of the GRP78 band was validated by Western blot analysis of cell lysates from Vero E6-ACE2 and H1299 cells transfected with either control siRNA or siRNAs against GRP78, as well as cells overexpressing GRP78 (Fig. 1d and Supplementary Fig. 1a). Our results indicated that SARS-CoV-2 infection led to a temporal increase of GRP78 protein level in both Vero E6-ACE2 and H1299 cells, peaking at 36 hpi compared to mock-infected cells. Furthermore, we determined that at MOI of 0.5 and 3, GRP78 protein and mRNA levels increased to similar levels at 24 hpi (Fig. 1e), and a temporal increase of *GRP78* mRNA levels was observed in the infected cells (Supplementary Fig. 1b).

**Fig. 1:**
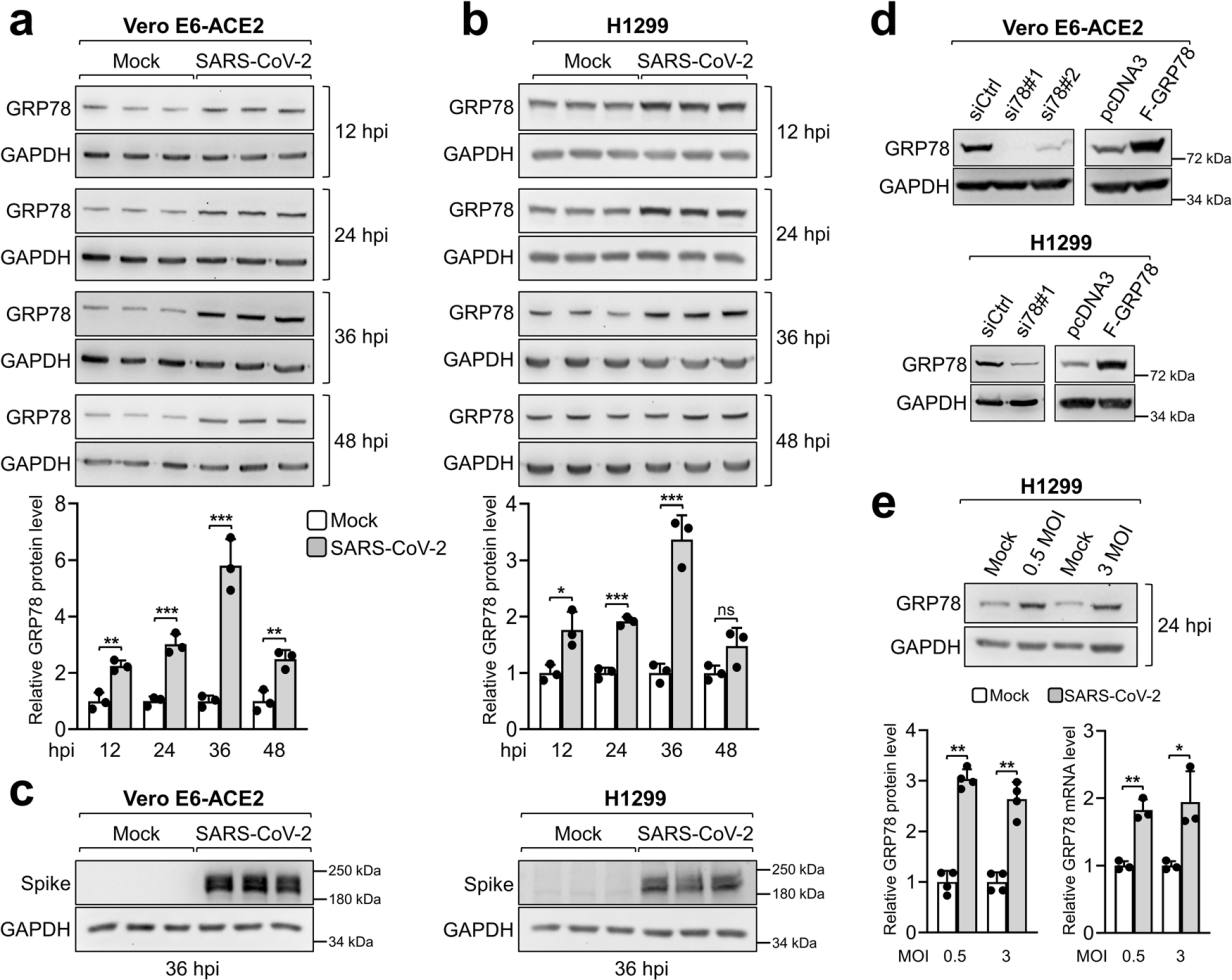
SARS-CoV-2 upregulated GRP78 protein and mRNA levels in infected cells. **a** Vero E6-ACE2 cells were mock-infected or infected with SARS-CoV-2 at an MOI of 0.5 in triplicate. The cells were collected at 12, 24, 36, and 48 hr post infection (hpi) and cell lysates were analyzed by western blot for GRP78 protein level with GAPDH serving as loading control. Quantitation of relative protein level of GRP78 normalized against GAPDH was shown in the graph below (*n* = 3). **b** Same as in **a** except the human lung epithelial cell line H1299 was used (*n* = 3). **c** Same as in **a** and **b** but 36 hpi cell lysates were analyzed by Western blot for SARS-CoV-2 Spike protein level with GAPDH serving as loading control (*n* = 3). **d** Vero E6-ACE2 (upper) or H1299 (lower) cells were transiently transfected with control siRNA or siRNAs against GRP78 (left panel) or empty vector pcDNA3 or vector expressing full-length Flag-tagged GRP78 protein (F-GRP78) (right panel) for 48 hr. Cell lysates were analyzed by Western blot for GRP78 protein level with GAPDH serving as loading control. **e** H1299 cells were mock-infected or infected with SARS-CoV-2 at an MOI of 0.5 or 3. The cells were collected at 24 hpi and cell lysates were analyzed by western blot (*n* = 4) or RT-qPCR (*n* = 3) for GRP78 protein or mRNA levels, respectively, with GAPDH serving as an internal control. Quantitation of relative protein (left) or mRNA (right) levels of GRP78 normalized against GAPDH was shown in the graphs below. Data are means ± S.E.M. of three repeats. *P < 0.05; **P < 0.01; ***P < 0.001; ns denotes not significant (Student’s *t* test). Source data are provided as a Source Data file.

Next, we knockdown GRP78 via siRNA and observed that in SARS-CoV-2 infected H1299 cells, decrease in GRP78 protein level resulting in a decrease in viral Spike protein level and production of infectious virions as determined by plaque assay (Fig. 2a–c). Similar results were observed for Vero E6-ACE2 cells (Supplementary Fig. 1c–e). Furthermore, treatment of Vero E6-ACE2 cells with HA15, a small molecule that binds GRP78 and inhibits its ATPase activity^15^, resulted in a dose-dependent reduction in both the size and numbers of SARS-CoV-2 plaques formed, without affecting cell viability (Fig. 2d, e, Supplementary Fig. 1f). Our results are consistent with another study where a different small molecule inhibitor of GRP78 suppressed the production of infectious SARS-CoV-2 virions^10^. Finally, HA15 treatment reduced the viral RNA level by 10-fold in the lungs of K18-hACE2 transgenic mice infected with SARS-CoV-2 (Fig. 2f). Collectively, these results showed that GRP78 knockdown or inhibition of its activity suppressed SARS-CoV-2 replication and infectivity in vitro and in vivo.

**Fig. 2:**
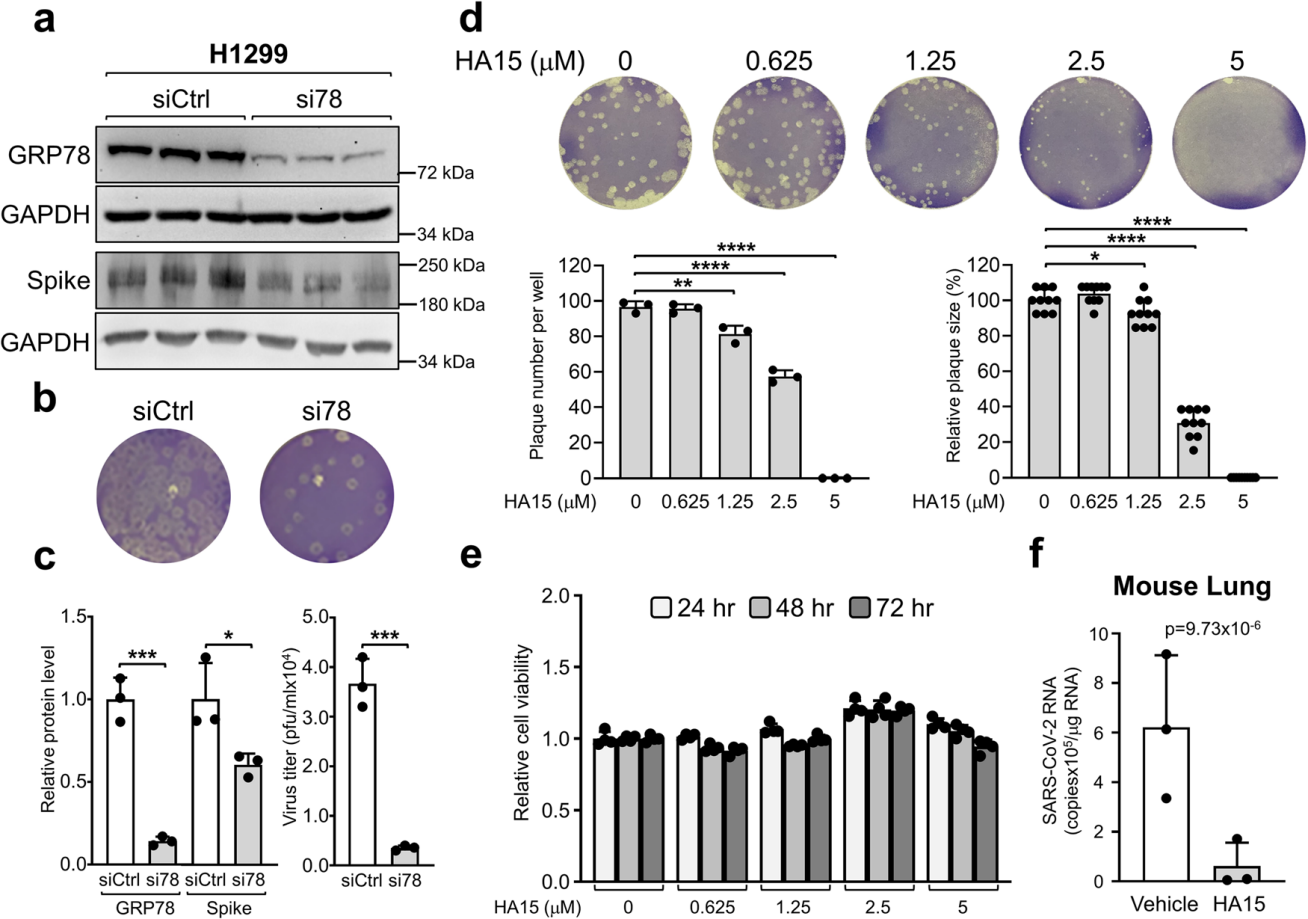
GRP78 knockdown by siRNA inhibited SARS-CoV-2 replication in vitro and GRP78 inhibitor HA15 blocked SARS-CoV-2 infection in vitro and in vivo. **a** H1299 cells were transiently transfected with control siRNA (siCtrl) or siRNA against GRP78 (si78#1) for 24 hr before infection with SARS-CoV-2 at an MOI of 3 for 24 hr. The cell lysates were analyzed by western blot for GRP78 and Spike protein levels with GAPDH serving as a loading control (*n* = 3). **b** The supernatant containing the newly released virions from **a** was collected and virus titer was determined by plaque assay (*n* = 3). **c** Quantitation of the relative protein levels of GRP78 and Spike normalized against GAPDH was shown in the graphs on the left and virus titer (pfu/ml) was shown in the graph on the right. **d** Confluent monolayers of Vero E6-ACE2 cells in six-well plates were infected with SARS-CoV-2 virus and treated with different concentrations of HA15 as indicated for 72 hr. At the end of treatment, the cells were stained with 0.2% crystal violet. The images are representatives of three repeats. Plaques were counted and plotted in the graph on the left (*n* = 3). Plaque size was measured and expressed relative to DMSO-treated control in the graph on the right (*n* = 10). **e** Vero E6-ACE2 cells cultured under identical conditions as in **d** were treated with DMSO or increasing concentration of HA15 from 0.625 μM to 5 μM and cell viability was measured by WST-1 assay at 24, 48, and 72 hr after drug treatment (*n* = 4). **f** K18-hACE2 transgenic mice infected with SARS-CoV-2 were treated with vehicle control or HA15. Three days post infection, lung tissue RNA was isolated, and RT-qPCR was performed in triplicate reactions to detect SARS-CoV-2 N protein sequence (*n* = 3). Data are means ± S.E.M. of three repeats. *P < 0.05; **P < 0.01; ***P < 0.001; ****P < 0.0001 (Student’s *t* test). Source data are provided as a Source Data file.

In conclusion, GRP78 is a pro-viral protein that is upregulated during SARS-CoV-2 infection, as observed here and in other studies in vitro^10–12^ and in patient tissues and serum^13,14^. Thus, we hypothesize that anti-GRP78 agents in combination with anti-SARS-CoV-2 therapeutics, could further suppress SARS-CoV-2 infection since GRP78 inhibition can deprive the virus of an essential chaperone for their entry and viral protein production. Whereas partial GRP78 expression is sufficient for normal cell homeostasis and survival^16^, GRP78 was highly expressed in adipose tissue and increased in older and obese diabetic human subjects^17^. Thus, the reduction of GRP78 has been proposed as a potential therapeutic target for reducing the severe progression and outcome of COVID-19 in older and obese diabetic patients^17^. These preclinical concepts warrant vigorous investigations in vivo.

## Methods

### Cell culture and drug treatment

The African green monkey kidney epithelial cell line Vero E6-ACE2 was cultured in Dulbecco’s modified Eagle medium (DMEM) containing 10% fetal bovine serum (FBS; Gemini Bio), 1% penicillin/streptomycin (pen/strep) (Corning Inc.), and 1 μg/ml puromycin. The human non-small cell lung adenocarcinoma cell line H1299 was cultured in RPMI-1640 medium containing 10% FBS and 1% pen/strep. All cell lines were maintained at 37 °C in a humidified atmosphere of 5% CO_2_ and 95% air. HA15 was purchased from Sigma Aldrich and dissolved in DMSO. Vero E6-ACE2 cells were treated with HA15 at indicated concentrations and 1% DMSO was used as a control. The Vero E6 and H1299 cell lines were obtained from ATCC.

### Virus propagation

The following reagent was deposited by the Centers for Disease Control and Prevention and obtained through BEI Resources, NIAID, NIH: SARS-Related Coronavirus 2, Isolate USA-WA1/2020, NR-52281. The virus was propagated in Vero E6-ACE2 in a DMEM media supplemented with 10% FBS, 1% penicillin-streptomycin (Gibco) and 0.5 µg/ml TPCK-treated trypsin (Worthington Biochemical). When 90% CPE was confirmed, supernatant was collected and passed through a 0.45 μm pore size polyethersulfone filter and aliquoted and stored at −80 °C until further use. The virus titer was determined by plaque assay.

### Plaque reduction assay

Evaluating the anti-viral activities of HA15 was done by plaque reduction assay as described previously^18^. Confluent monolayers of Vero E6-ACE2 cells in 6-well plates were washed once with DMEM and infected with approximately 100 plaque-forming units (PFUs) of SARS-CoV-2 in each well. The plates were incubated in 33 °C for 45 min for virus adsorption. The virus inoculum was then removed and replaced by overlay media (DMEM containing 1% low-melting agarose without serum) containing 2-fold serial dilutions of HA15 and placed in 33 °C CO_2_ incubator for 72 hr when plaques can be visualized under light. The cells were fixed with 4% formaldehyde solution for at least 30 min and the overlaid agarose was removed. The cells were stained with 0.2% (w/v) crystal violet solution. The plaques were counted by visual examination and the size of the plaques were measured by scale loupe.

### Virus infection and cell harvest

Vero E6-ACE2 and H1299 cells were seeded in 6-well plates and allowed to attach. Cells were washed once with fresh DMEM and infected with 0.5 or 3 MOI of SARS-CoV-2 in each well. DMEM media was used as mock infection. The plates were incubated on a rocker in 33 °C for 45 min for virus adsorption. The virus inoculum was then removed and replaced by DMEM media and placed in 33 °C CO_2_ incubator. The cell pellets were collected at indicated time points and stored at −80 °C. The cell pellet samples were lysed, and cell lysates were subjected to Western blot analysis or RT-qPCR for the detection of the proteins or RNA of interest.

### Transfection of siRNAs and plasmid DNA expression vector

For siRNA knockdown, the cells were transfected with Lipofectamine RNAiMAX reagent (Thermo Fisher Scientific) with 60pmol of siRNAs (Dharmacon-GE Healthcare) according to the manufacturer’s recommendation. The control siRNA and siRNAs targeting GRP78 have been previously described^19^ and their sequences are as follows: siCtrl: GAGAUCGUAUAGCAACGGUdTdT; si78#1: GGAGCGCAUUGAUACUAGAdTdT; si78#2: CUUAAGUCUCGAAUGUAAUdTdT. The construction of the expression plasmid for FLAG-GRP78 (F-GRP78) has been described previously^20^. The empty vector pcDNA3 or the expression vector for F-GRP78 was transfected into Vero E6-ACE2 and H1299 cells using the BioT Transfection Reagent (Bioland Scientific) following the manufacturer’s recommendation. The cells were incubated with the transfection mix for 48 hr before harvesting cells for immunoblot analysis of the protein of interest.

### Immunoblot analysis

Preparation of cell lysates and immunoblot analysis has been described previously^21^. Proteins were electrophoresed in 8% or 10% SDS-PAGE gels, transferred to Nitrocellulose membrane, and probed with the following antibodies. Primary antibodies: mouse anti-GRP78 (1:1000, BD Biosciences, 610979), mouse anti-GAPDH (1:1000, Santa Cruz Biotechnology, Inc., sc-32233), mouse anti-SARS-CoV-2 Spike Protein (S1-NTD) (1:1000, Cell Signaling, #42172). Secondary antibody: mouse IgG1 binding protein conjugated to HRP (1:1000, Santa Cruz Biotechnology, Inc., sc-525408). HRP signal was detected by SuperSignal West Pico Chemiluminescence substrate (Thermo Fisher Scientific, 34080) and protein bands were visualized by ChemiDoc XRS + imager (Bio-Rad Laboratories) and quantified by Image Lab software version 4.0.1 build 6 (Bio-Rad Laboratories).

### RNA extraction and reverse transcription quantitative real-time PCR

Vero E6-ACE2 cells were mocked infected or infected with SARS-CoV-2 at 0.5 or 3 MOI for 12 or 24 hours. The cells were lysed with TRI reagent (Millipore-Sigma, Burlington, MA) and total RNA was extracted according to the manufacturer’s recommendations. 1 μg of total RNA was used to synthesized cDNA using the qScript cDNA Supermix (QuantaBio, Beverly, MA) following manufacturer’s protocol. Quantitative real-time PCR analysis of *GRP78* and *GAPDH* mRNA was conducted using the KAPA SYBR FAST qPCR Master Mix (Roche Sequencing and Life Science, Wilmington, MA) and analyzed by the Stratagene MX3000P Real-Time QPCR System (Agilent, Santa Clara, CA). The PCR conditions are 40 cycles, 15 s at 95 °C, 15 s at 60 °C, 15 s at 72 °C. The sequences for the primers used in this study are as followed: *GRP78* 5′-GTCAGGCGATTCTGGTCATT-3′ and 5′-GGTGAAAGACCCCTGACAAA-3′, *GAPDH* 5′- TGCACCACCAACTGCTTAGC-3′ and 5′-GGCATGGACTGTGGTCATGAG-3′.

### WST-1 cell viability assay

Vero E6-ACE2 cells were seeded at a density of 10,000 cells per wells in a 96-well plate with DMEM media containing 10% FBS and 1% pen/strep. The cells were allowed to attached overnight and next day the media was removed and replaced with DMEM media containing 1% pen/strep and no FBS. The cells were then treated with DMSO or increasing concentrations of HA15 (0.625 μM to 5 μM). Cell viability was measured at 24, 48, and 72 hr post drug treatment using the (4-[3-(4-Iodophenyl)-2-(4-nitro-phenyl)-2H-5-tetrazolio]-1,3-benzene sulfonate) WST-1 cell proliferation assay kit (Takara Bio USA, Inc., San Jose, CA) according to manufacturer’s recommendation. Colorimetric measurement was detected using a Model 680 Microplate Reader (Bio-Rad Laboratories, Hercules, CA) at a wavelength of 450 nm and subtracted by the reference wavelength of 650 nm. Background absorbance of blank media (DMEM 1% pen/strep) was also measured and subtracted from the sample reading.

### Animals and in vivo procedures

K18-hACE2 transgenic mice were purchased from Jackson Laboratory (cat. no. 034860). Female mice (about 10-week-old) were used. All animal care and experiments were performed according to the NIH guidelines for the care and use of laboratory animals. All animal studies were approved by the Institutional Animal Care and Use Committee of USC. Mice (*n* = 3 for each group) were intranasally infected with 10^3^ pfu of SARS-CoV-2 virus (USA-WA1/2020) plaque isolate in 30 μl PBS. HA15 (Cat. #CS-5825) was obtained from ChemScene (Monmouth Junction, NJ, USA). HA15 was first dissolved in DMSO and diluted in PBS for intraperitoneal injection. HA15 was intraperitoneally injected daily at 35 mg/kg from the time infected to day 3 post infection. On day 3, mice were euthanized by isoflurane overdose and the lung tissues were collected, homogenized, and lysed in 500 μl of TRIzol for RNA isolation and RT-qPCR analysis.

### RNA extraction and RT-qPCR analysis of mouse lung tissues

Total RNA was isolated from single-cell suspended mouse tissue in 500 μl of Trizol (Thermo Fisher Scientific, Waltham, MA). RNA was extracted following the manufacturer’s protocol for the Monarch Total RNA miniprep kit (New England Biolabs, Ipswich, MA). Complementary DNA (cDNA) was generated using 100 ng of total RNA with the iScript Reverse Transcription Supermix (Bio-Rad Laboratories) following the manufacturer’s protocol. qPCR was performed in a 96-well plate with PrimeTime® Gene Expression Master Mix (Integrated DNA Technologies (IDT), Coralville, IA) in triplicate with 1 μl of cDNA using primers for SARS-CoV-2 N1 protein and murine β-actin on the Bio-Rad CFX96 Real-Time PCR Detection system. Relative gene expression of SARS-CoV-2 N protein was calculated based on a genomic equivalent (GE) standard curve using the 2019-nCoV_N_Positive Control DNA (catalog# 10006625, IDT). The GE from the experimental samples was derived from this curve using the Maestro analysis software. Pre-designed TaqMan probes were purchased from IDT: N1 [SARS-CoV-2 (2019-nCoV) CDC RUO Primers and Probes Cat# 10006713], murine β-actin (Cat# Mm.PT.39a.22214843.g).

### Statistical analysis

All pair-wise comparisons were made using the two-tailed unpaired Student’s *t* test in Microsoft Excel. Data are presented as the mean ± Standard Error of the mean (S.E.M.). A *p* value of ≤0.05 is considered statistically significant. All graphs were generated with GraphPad Prism v.8.3.0 (GraphPad Software, San Diego, CA).

### Reporting summary

Further information on research design is available in the Nature Research Reporting Summary linked to this article.

## Supplementary information

Supplementary information

Reporting Summary

## Data Availability

All data generated and analyzed during this study are included in this published article and its supplementary information files. Source data are provided with this paper.

## Source data

Source Data
