# Supplementary material for: The stress-inducible ER chaperone GRP78/BiP is upregulated during SARS-CoV-2 infection and acts as a pro-viral protein
Source: Nat Commun. 2022 Nov 14;13:6551. doi: 10.1038/s41467-022-34065-3 (PMC9663498; doi:10.1038/s41467-022-34065-3)
Supplement: Supplementary file 1 — Supplementary information [file 41467_2022_34065_MOESM1_ESM.pdf]

Supplementary Information

The stress-inducible ER chaperone GRP78/BiP is upregulated during SARS-CoV-2 infection and acts as a pro-viral protein

Woo-Jin Shin[1#], Dat P. Ha[2,4#], Keigo Machida[3] and Amy S. Lee[2,4*]

[1]Florida Research and Innovation Center, Cleveland Clinic, Port St. Lucie, Florida, USA, [2]Department of Biochemistry and Molecular Medicine, [3]Department of Molecular Microbiology and Immunology, University of Southern California, Keck School of Medicine, Los Angeles, California, USA and [4]USC Norris Comprehensive Cancer Center

*Corresponding author: Amy S. Lee, Ph.D., Department of Biochemistry and Molecular Medicine, University of Southern California, Keck School of Medicine, USC Norris Comprehensive Cancer Center, 1441 Eastlake Avenue, Los Angeles, CA 90089. [Email] amylee@usc.edu.

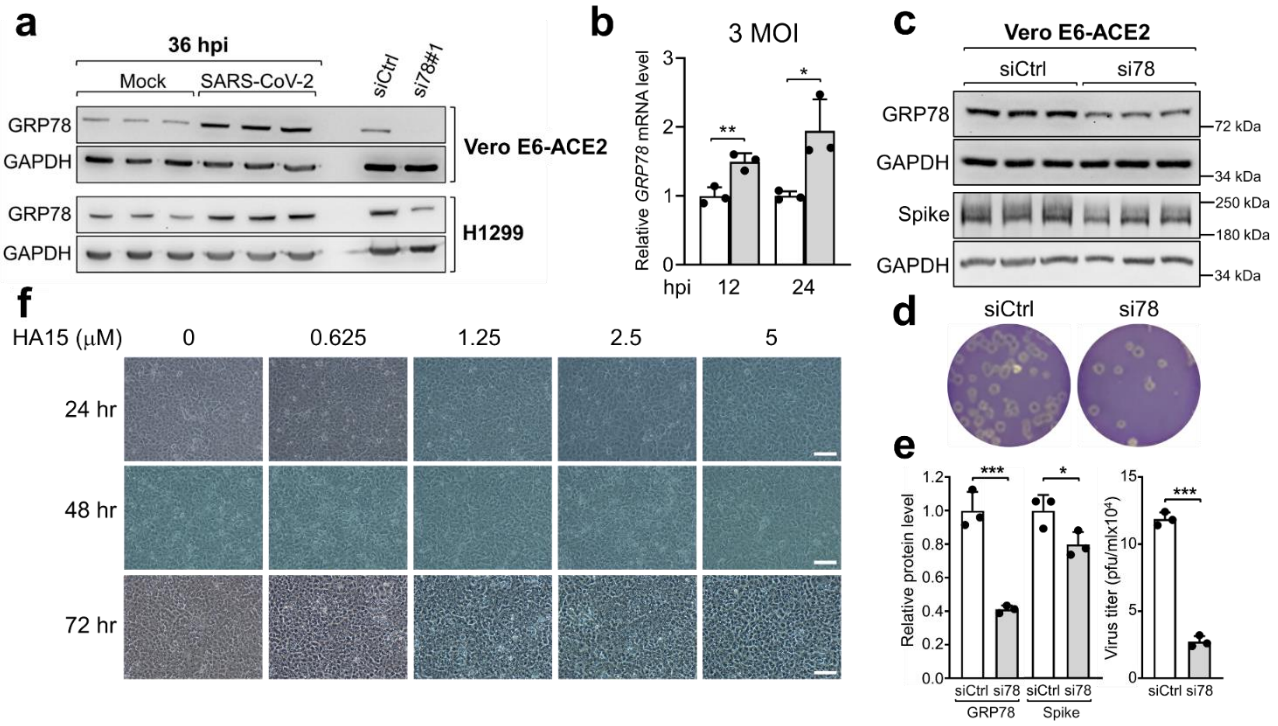

Supplementary Fig. 1. SARS-CoV-2 infection up-regulated GRP78 protein and mRNA levels and GRP78 knockdown by siRNA inhibited SARS-CoV-2 replication *in vitro* while HA15 treatment for up to 72 hr did not affect cell viability of Vero E6-ACE2 cells. (**a**) The cell lysates (36 hpi) from **Fig. 1a,b** were analyzed by Western blot along with Vero E6-ACE2 or H1299 cells transiently transfected with control siRNA (siCtrl) or siRNA against GRP78 (si78#1) to demonstrate the specificity of the observed GRP78 protein bands. GAPDH serves as loading control. (**b**) H1299 cells were mock-infected or infected with SARS-CoV-2 at an MOI of 3. The cells were collected at 12 and 24 hpi and total RNA were analyzed by RT-qPCR for *GRP78* mRNA level with *GAPDH* mRNA serving as internal control. Quantitation of the relative mRNA level of *GRP78* normalized against *GAPDH* was shown in the graph (n=3). (**c**) Vero E6-ACE2 cells were transiently transfected with control siRNA (siCtrl) or siRNA against GRP78 (si78#1) for 24 hr before infection with SARS-CoV-2 at an MOI of 3 for 24 hr. The cell lysates were analyzed by Western blot for GRP78 and Spike protein levels with GAPDH serving as loading control (n=3). (**d**) The supernatant containing the newly release virions from (**c**) was collected and virus titer was

determined by plaque assay (n=3). (**e**) Quantitation of the relative protein levels of GRP78 and Spike normalized against GAPDH was shown in the graphs on the left and virus titer (pfu/ml) was shown in the graph on the right. (**f**) Vero E6-ACE2 cells were treated with DMSO or increasing concentration of HA15 from 0.625μM to 5μM for the indicated times as in **Fig. 2e** but brightfield microscopy images of the cells were taken. Scale bars represent 100μm. Data are means ± S.E.M. of three repeats. *, $P<0.05$; **, $P<0.01$; ***, $P<0.001$ (Student's *t* test). Source data are provided as a Source Data file.
