# Supplementary material for: The stress-inducible ER chaperone GRP78/BiP is upregulated during SARS-CoV-2 infection and acts as a pro-viral protein
Source: Nat Commun. 2022 Nov 14;13:6551. doi: 10.1038/s41467-022-34065-3 (PMC9663498; doi:10.1038/s41467-022-34065-3)
Supplement: Supplementary file 2 — Reporting Summary [file 41467_2022_34065_MOESM2_ESM.pdf]

nature portfolio

Corresponding author(s): Amy S. Lee

Last updated by author(s): Sep 21, 2022

Reporting Summary

Nature Portfolio wishes to improve the reproducibility of the work that we publish. This form provides structure for consistency and transparency in reporting. For further information on Nature Portfolio policies, see our Editorial Policies and the Editorial Policy Checklist.

Statistics

For all statistical analyses, confirm that the following items are present in the figure legend, table legend, main text, or Methods section.

n/a	Confirmed	
☐	☒	The exact sample size (*n*) for each experimental group/condition, given as a discrete number and unit of measurement
☐	☒	A statement on whether measurements were taken from distinct samples or whether the same sample was measured repeatedly
☐	☒	The statistical test(s) used AND whether they are one- or two-sided<br>*Only common tests should be described solely by name; describe more complex techniques in the Methods section.*
☒	☐	A description of all covariates tested
☒	☐	A description of any assumptions or corrections, such as tests of normality and adjustment for multiple comparisons
☐	☒	A full description of the statistical parameters including central tendency (e.g. means) or other basic estimates (e.g. regression coefficient) AND variation (e.g. standard deviation) or associated estimates of uncertainty (e.g. confidence intervals)
☐	☒	For null hypothesis testing, the test statistic (e.g. *F*, *t*, *r*) with confidence intervals, effect sizes, degrees of freedom and *P* value noted<br>*Give P values as exact values whenever suitable.*
☒	☐	For Bayesian analysis, information on the choice of priors and Markov chain Monte Carlo settings
☒	☐	For hierarchical and complex designs, identification of the appropriate level for tests and full reporting of outcomes
☒	☐	Estimates of effect sizes (e.g. Cohen's *d*, Pearson's *r*), indicating how they were calculated

Our web collection on statistics for biologists contains articles on many of the points above.

Software and code

Policy information about availability of computer code

Data collection	Not Applicable
Data analysis	Not Applicable

For manuscripts utilizing custom algorithms or software that are central to the research but not yet described in published literature, software must be made available to editors and reviewers. We strongly encourage code deposition in a community repository (e.g. GitHub). See the Nature Portfolio guidelines for submitting code & software for further information.

Data

Policy information about availability of data

All manuscripts must include a data availability statement. This statement should provide the following information, where applicable:

- Accession codes, unique identifiers, or web links for publicly available datasets
- A description of any restrictions on data availability
- For clinical datasets or third party data, please ensure that the statement adheres to our policy

All data generated and analysed during this study are included in this published article and its supplementary information files.

Field-specific reporting

Please select the one below that is the best fit for your research. If you are not sure, read the appropriate sections before making your selection.

☒ Life sciences ☐ Behavioural & social sciences ☐ Ecological, evolutionary & environmental sciences

For a reference copy of the document with all sections, see nature.com/documents/nr-reporting-summary-flat.pdf

Life sciences study design

All studies must disclose on these points even when the disclosure is negative.

Sample size	No sample-size calculation was performed. A sample size of n=3 is commonly used in biological studies using cell cultures.
Data exclusions	No data were excluded from the analyses
Replication	All experiments were performed in triplicate, independent biological repeats. All data were shown in the study.
Randomization	Not Applicable
Blinding	Not Applicable

Reporting for specific materials, systems and methods

We require information from authors about some types of materials, experimental systems and methods used in many studies. Here, indicate whether each material, system or method listed is relevant to your study. If you are not sure if a list item applies to your research, read the appropriate section before selecting a response.

Materials & experimental systems

n/a	Involved in the study	
☐	☒	Antibodies
☐	☒	Eukaryotic cell lines
☒	☐	Palaeontology and archaeology
☐	☒	Animals and other organisms
☒	☐	Human research participants
☒	☐	Clinical data
☒	☐	Dual use research of concern

Methods

n/a	Involved in the study	
☒	☐	ChIP-seq
☒	☐	Flow cytometry
☒	☐	MRI-based neuroimaging

Antibodies

Antibodies used	Primary antibodies: mouse anti-GRP78 (1:1000, BD Biosciences, 610979), mouse anti-GAPDH (1:1000, Santa Cruz Biotechnology, Inc., sc-32233), mouse anti-SARS-CoV-2 Spike Protein (S1-NTD) (1:1000, Cell Signaling, #42172). Secondary antibody: mouse IgG1 binding protein conjugated to HRP (1:1000, Santa Cruz Biotechnology, Inc., sc-525408).
Validation	All the antibodies used were obtained from commercial sources and their specificity was validated by each manufacturer. In addition, using cell lysates in Western blots, we validated the specificity of the anti-GRP78 antibody by siRNA knock-down and overexpression of GRP78.

Eukaryotic cell lines

Policy information about cell lines

Cell line source(s)	The Vero E6 and H1299 cell lines were obtained from ATCC.
Authentication	These commercially obtained cell lines were authenticated by vendors.
Mycoplasma contamination	None
Commonly misidentified lines (See ICLAC register)	None

Animals and other organisms

Policy information about studies involving animals; ARRIVE guidelines recommended for reporting animal research

Laboratory animals	K18-hACE2 transgenic mice were purchased from Jackson Laboratory (cat. no. 034860)
Wild animals	*Provide details on animals observed in or captured in the field; report species, sex and age where possible. Describe how animals were caught and transported and what happened to captive animals after the study (if killed, explain why and describe method; if released, say where and when) OR state that the study did not involve wild animals.*
Field-collected samples	*For laboratory work with field-collected samples, describe all relevant parameters such as housing, maintenance, temperature, photoperiod and end-of-experiment protocol OR state that the study did not involve samples collected from the field.*
Ethics oversight	All animal studies were approved by the Institutional Animal Care and Use Committee of the USC

Note that full information on the approval of the study protocol must also be provided in the manuscript.
